# A comparison of the substance use related risk and protective factor profiles for American Indian and White American youth: a mixed studies review

**DOI:** 10.3389/fpubh.2024.1046655

**Published:** 2024-01-31

**Authors:** Melanie Nadeau, Kathryn Wise, Revathi B. Sabbella, Devon Olson

**Affiliations:** ^1^Department of Indigenous Health, School of Medicine and Health Sciences, University of North Dakota, Grand Forks, ND, United States; ^2^School of Medicine and Health Sciences, University of North Dakota, Grand Forks, ND, United States

**Keywords:** American Indian, non-Hispanic White American, substance use, risk factor, protective factor, youth

## Abstract

**Background:**

American Indian youth are disproportionately impacted by substance use compared to White American youth in the United States. This mixed studies review focused on gathering data to examine the similarities and differences between the risk and protective factor profiles for substance use among American Indian and White American youth aged 10–21.

**Methods:**

A scan of the existing literature was needed to review substance use related risk and protective factors for American Indian and White American youth. Search phrases were created to ensure maximum relevant results from existing literature through 2021. After deduplication, an appraisal tool was utilized to review 343 records. A total of 19 articles were deemed relevant. Data from relevant articles was recorded and categorized into the levels of the Social Ecological Model.

**Results:**

Significant and salient risk and protective factors of substance use for both American Indian and White American youth presented at the individual, interpersonal (family/non-family), and community levels of the Social Ecological Model. A total of 84 factors were found from relevant articles, 55 risk factors and 29 protective factors. When comparing the American Indian and White American youth profiles, a total of 29 unique differences between American Indian (*n* = 21) and White American youth (*n* = 8) were identified.

**Discussion:**

Results from this review can be utilized to inform Tribal leaders, stakeholders, and policymakers, which will ultimately influence health intervention strategies and prioritizations. Given the limited evidence though, researchers should be responsive to Tribal communities’ call to action for utilizing a culturally rooted approach.

## Background

American Indian youth are disproportionately impacted by substance use compared to Non-Hispanic White Americans in the United States (1). Substance use appears to be high for American Indian youth compared to White American youth. However, the data are very scarce throughout the United States. Each year the Substance Abuse and Mental Health Services Administration (SAMHSA) conducts the National Survey of Drug Use and Health (NSDUH) survey which includes approximately 70,000 people aged 12 and older (2). This survey is conducted to track the trends in the use of alcohol, tobacco, and various types of drugs and identifies populations at high risk for substance use and misuse (1). In the 2018 survey, the total number of participants was 24,896: 12,957 being White American (52%), and only 159 being American Indian or Alaska Native (0.6%) (2). Given that there are over 6 million American Indians and Alaska Natives in the United States with 29% being under the age of 18 (3), meaningful comparisons between the two ethnic populations are difficult because of the lack of representative data for Tribal Nations.

Substance use is a contributor to a multitude of negative health outcomes. Substance use can lead to a multitude of co-occurring health issues such as lung or heart disease, stroke, cancer (4), or mental health conditions (5). Substances that are injected intravenously can increase the risk of contracting HIV (6), hepatitis B and C (7). Substances that are ingested orally, such as alcohol can cause harm to the digestive system (8) or smoking which can cause damage to the lungs and make the upper respiratory system more susceptible to infections (4, 9). Furthermore, when an individual struggles with substance abuse for a long period of time, the brain adapts. This can lead to changes in brain chemistry which can then lead to the development of physical dependence to the substance (10).

American Indian individuals experience disparate health outcomes when compared with other races and ethnicities in the United States (11). The leading causes of death for American Indians and Alaska Natives include heart disease, cancer, unintentional injuries, and diabetes (11). The life expectancy for American Indians and Alaska Natives is substantially lower at 73.0–78.5 years, which is 5.5 years lower than all other races and ethnicities in the United States (11). Caution should be used in the use of national data to inform initiatives focused on Tribal peoples as significant geographic differences exist. For example, Christensen et al. explored premature mortality patterns among American Indians in South Dakota from 2000 to 2010 and found that the median age of death in South Dakota for American Indians in 2010 was 58 years compared to 81 years for White Americans (12). American Indian populations also suffer disproportionately from cancer compared to all other races and ethnicities in the nation (13). In 2018, for every 100,000 American Indian and Alaska Native people, 259 new cancer cases were reported (13). American Indians and Alaska Natives experience colorectal, kidney, and stomach cancer at higher rates than non-Hispanic White Americans (14). According to a study that examined cancer trends in the United States for the years 2010–2015, incidence rates for the American Indian/Alaska Native population compared to the White American population were approximately 12% to 2.3 times higher for a multitude of cancers including lung, myeloma, colorectal cancer, stomach, kidney and liver (15). Additionally, from 2010–2015, the prevalence of any tobacco product use was higher among the American Indian and Alaska Native population, approximately 43%, compared to other racial/ethnic groups in the United States (16). Identification of the unique risk and protective factor profiles for substance use for American Indians is essential for addressing health disparities and reducing morbidity and mortality rates.

**Figure 1:**
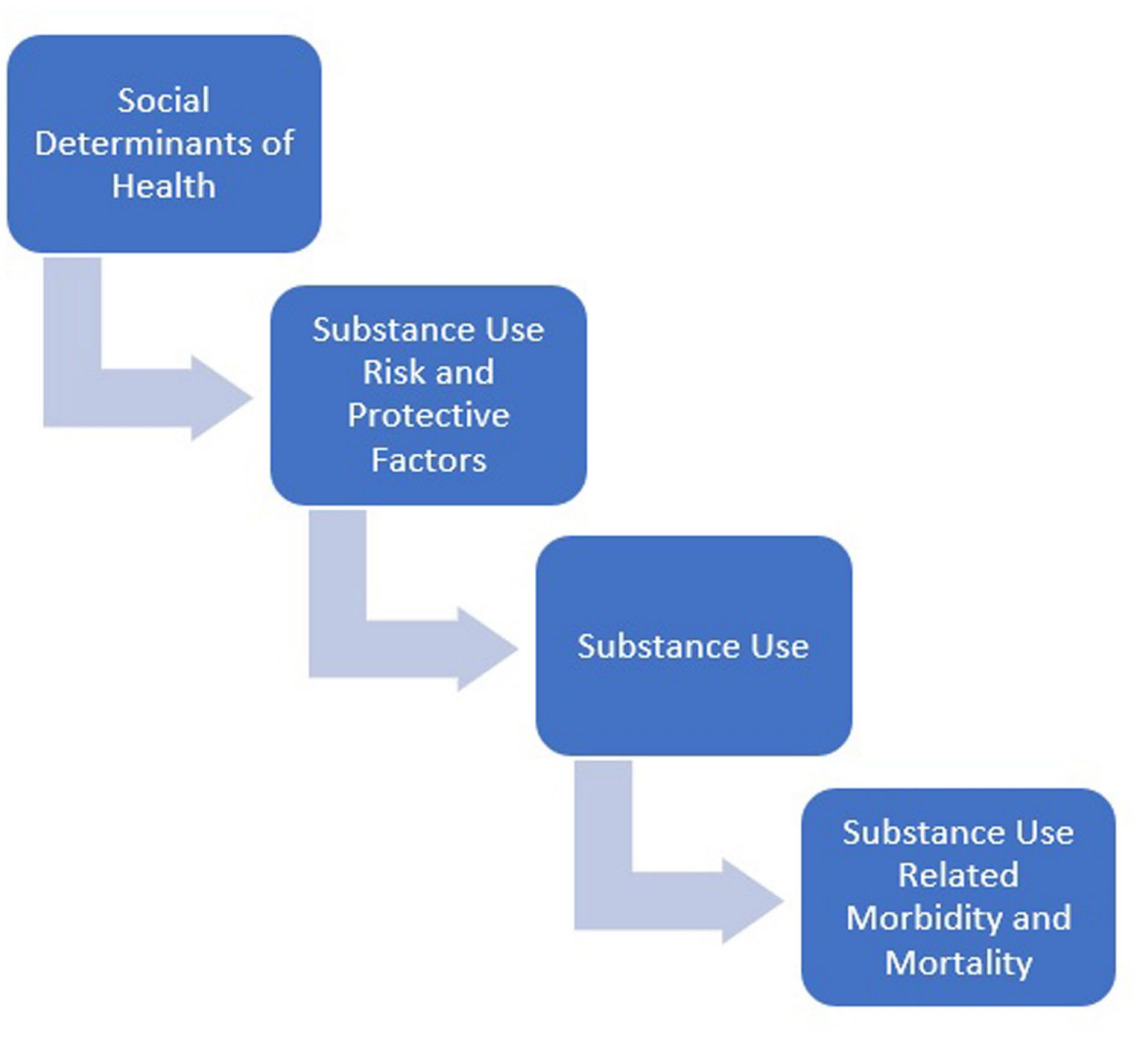
Concept model for substance use related risk and protective factors.

More broadly, a multitude of factors impact health and wellbeing including social, economic, physical, environmental, individual health behavior, clinical care, and genetics. Social, economic, and physical environmental factors, better known as the Social Determinants of Health, contribute to the wide health disparities and inequities that exist today. According to Healthy People 2030, “Social Determinants of Health are the conditions in the environments where people are born, live, learn, work, play, worship, and age that affect a wide range of health, functioning, and quality of life outcomes and risk” (17). In general, some Social Determinants of Health for the US population include income level, educational opportunities, occupation or employment status, gender inequity, racial segregation, food insecurity, and access to housing (17). Social Determinants of Health that are specific to American Indian populations include but are not limited to genocide, assimilation, racism, poverty, unequal access to health care, lack of education, stigma, and the purposeful destruction of lands, languages, and traditional practices (18). According to Healthy People, there are numerous factors that are associated with substance use including biological, social, environmental, psychological, genetic, interpersonal, household, and community factors (19). Figure 1 illustrates how the Social Determinants of Health and substance use related risk and protective factors can lead to substance use related morbidity and mortality.

According to the US Census, approximately 71% of American Indians and Alaska Natives live in urban areas across the United States (20). This large percentage accounts for those who moved or were forced to relocate to urban areas because of government policy, lack of economic and educational opportunities, and limited access to healthcare and other services (21). Although many American Indians reside in urban areas, only 25% of them reside in counties served by Urban Indian Health programs (20). Even in urban areas, American Indian populations still suffer from health disparities at disproportionate rates compared to other racial and ethnic groups including chronic disease, infectious disease, and unintentional injury with extraordinarily high levels of co-morbidity and mortality (20). American Indians who live in urban areas may also experience unique hardships due to weakened tribal ties and sense of community. The Indian Relocation Act of 1956 encouraged American Indians to move to urban locations where jobs were more plentiful (21). Many of these individuals and or their families never returned to the reservation (21).

This literature review focused on gathering data to examine the similarities and differences between the risk and protective factor profiles for substance use among American Indian and White American youth. Data gathered from relevant articles was organized utilizing the Social Ecological Model (SEM) and placed at the correct level; individual-, interpersonal- (family and non-family), community-, institutional-, policy-, and cultural-. The SEM illustrates that health is affected by the intersection between all levels of the model. Moreover, this model suggests that to prevent a health outcome, it is necessary to act across multiple levels of the model. Results from this review can be utilized to inform Tribal leaders, stakeholders, and policymakers on the risk and protective factor profiles for substance use for American Indian youth compared with White American youth, which can influence health intervention strategies and prioritizations. Additionally, when working with urban populations, the comparison between these two populations can identify strengths and limitations in current health interventions as well as illustrate ways to create protective factor rich environments that are more inclusive. Given culturally rooted evidence-based programs specific to American Indian youth that are applicable across the SEM are often non-existent, examination of the unique health profiles from both populations can influence health programs specific to the population, allowing health practitioners to be more responsive, moving away from a one size fits all approach.

## Methods

To gain insight on the risk and protective factor profiles for both American Indian and White American youth, a thorough search through existing literature was needed. This literature review examined qualitative and quantitative peer reviewed studies through 2021 to identify substance use related risk and protective factors for American Indian and White American youth aged 10–21. The search phrases were created by a medical librarian (author Olson) to ensure maximum results of available articles for review. A sample search phrase, the search phrase for PubMed is included in the Supplementary material. Databases were chosen based on their subject coverage and accessibility via the authors’ institutional library. PubMed and CINAHL were selected for their comprehensive indexing of medical and health sciences literature, which address substance abuse via the lenses of those disciplines. PsycINFO and ERIC were also searched to illuminate psychosocial and community impacts of Indigenous adolescent substance abuse, as well as the role of educational institutions, where this population spends most of their time. The following databases were utilized: PubMed (*n* = 197), ERIC (*n* = 64), CINAHL (*n* = 239), PsychInfo (*n* = 102) for a total of 602 articles. Author Olson removed duplicates (*n* = 259), which resulted in a total of 343 articles which were imported into Covidence. No additional duplicates were identified by Covidence. Covidence is an online systematic review management tool.

**Figure 2:**
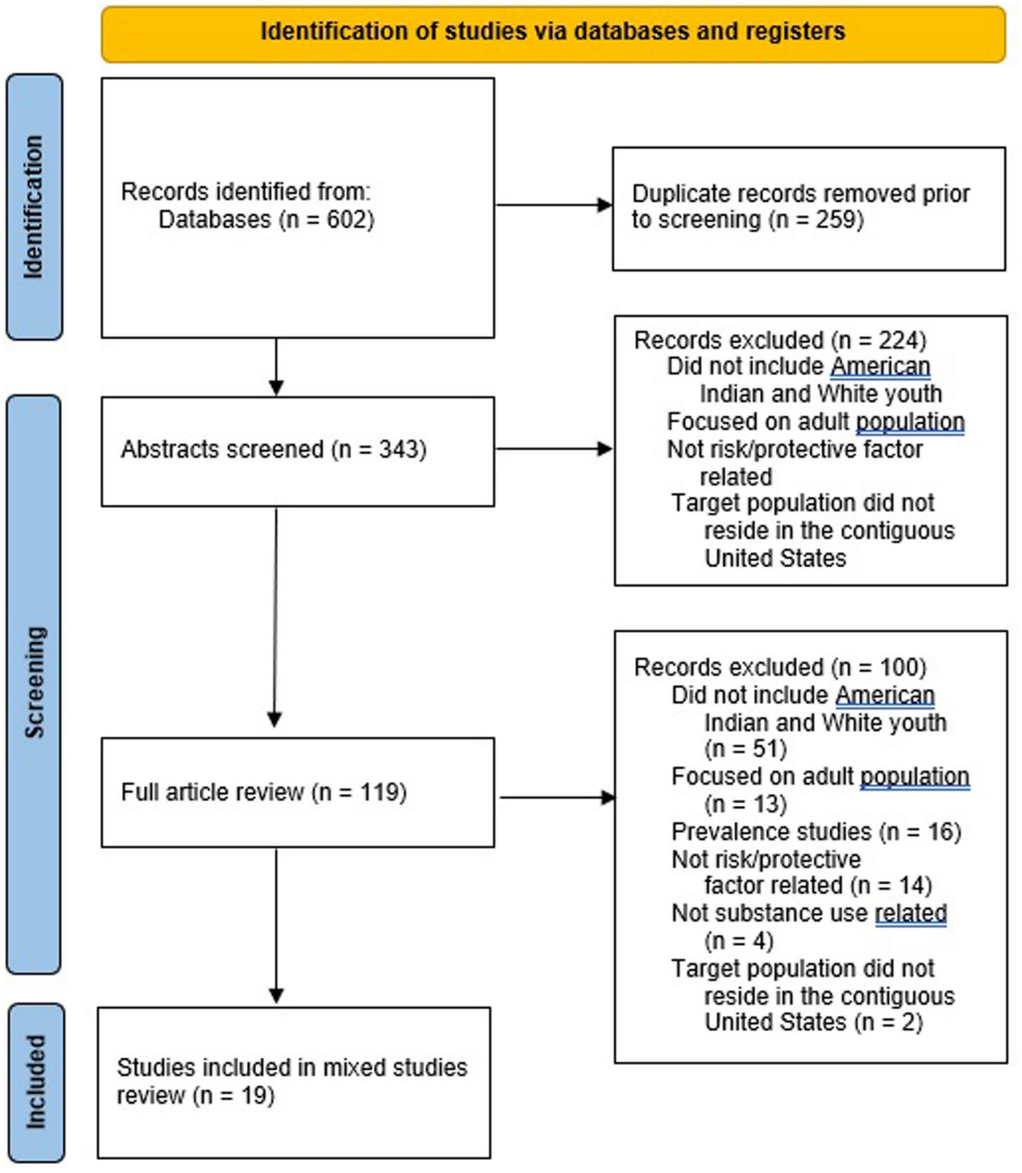
PRISMA diagram.

Once added to Covidence, abstracts and titles were screened followed by full-text screening. Both reviewers had to agree on inclusion or exclusion of records and the reasoning behind exclusion. The PRISMA diagram in Figure 2 illustrates article exclusion and inclusion results. Abstract review was conducted for 343 records. Abstract records were excluded (*n* = 224) if they did not meet the following criteria: did not include American Indian and White American youth, focused on the adult population, not risk/protective factor related, and target population did not reside in the contiguous United States. Full-text review was conducted for the remaining 119 records. The Mixed Methods Appraisal Tool (MMAT) was utilized by both reviewers to appraise the quality of articles before data extraction. This tool was designed for systematic mixed studies reviews (i.e., reviews that include qualitative, quantitative, and mixed methods studies). Records were excluded (*n* = 100) if they did not meet the following criteria: did not include American Indian and White American youth (*n* = 51), focused on the adult population (*n* = 13), prevalence studies (*n* = 16), not risk/protective factor related (*n* = 14), not substance use related (*n* = 4), and the target population did not reside in the contiguous United States (*n* = 2). After review, 19 studies were included for data extraction. Publication years for these articles ranged from 1976 to 2021. Significant risk and protective factors were organized into the levels of the SEM utilizing Excel. Substance use related risk and protective factor findings from the literature are described in the results section.

## Results

After extracting significant and salient substance use related risk and protective factors from relevant articles, data was organized into the levels of the SEM. Risk and protective factors presented at the following levels: individual; interpersonal (family and non-family); and community. No risk or protective factors were identified at the institutional-, policy- or cultural-level.

Overall, 84 factors were found, 55 risk factors and 29 protective factors. Approximately 65% of the factors found were risk factors. Of the risk factors identified, 37 were at the individual level, 5 at the interpersonal (family) level, 10 at the interpersonal (non-family) level, and 3 at the community level. All identified risk factors are illustrated in Supplementary Table S1. Approximately 35% of the factors found were protective factors. Of the protective factors found, 16 were at the individual level, 11 at the interpersonal (family) level, 1 at the interpersonal (non-family) level, and 1 at the community level. All protective factors found are illustrated in Supplementary Table S2.

The following substances were identified in the literature review: 9 articles focused on multiple substances (i.e., marijuana, alcohol, and cigarette use), 5 articles focused on alcohol use, 1 article focused on methamphetamine use, 1 focused on marijuana use, and 3 focused on inhalant use. Inhalants are defined as “volatile substances that produce chemical vapors that can be inhaled to induce a psychoactive, or mind-altering effect” (22). Some examples include “dry-cleaning fluids, degreasers, gasoline, glues, correction fluids, felt-tip markers,” and paint thinners and removers (22).

### Factors unique to American Indian and White American youth

**Figure 3:**
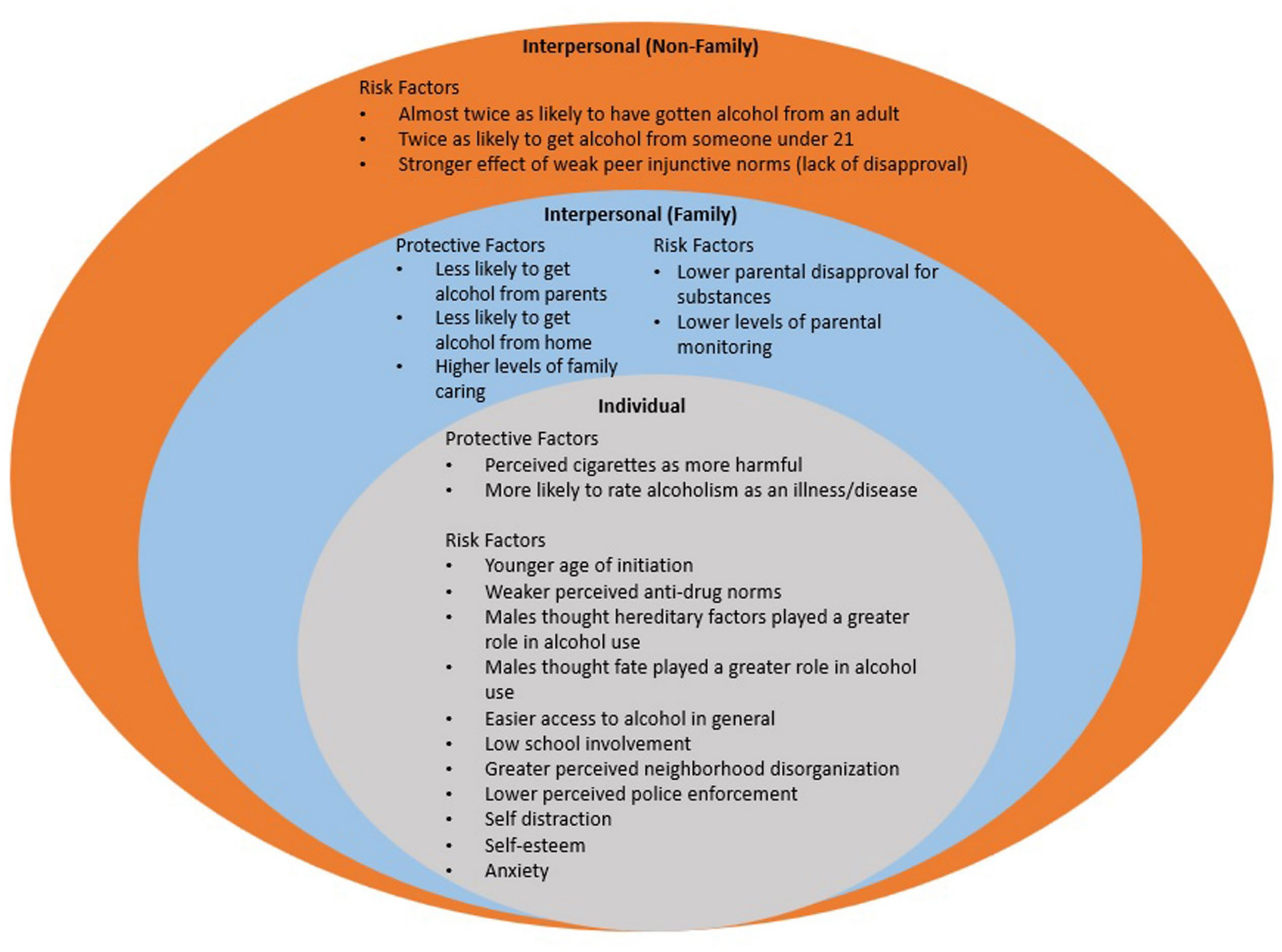
Risk and protective factors unique to american indian youth across the ecological model.

**Figure 4:**
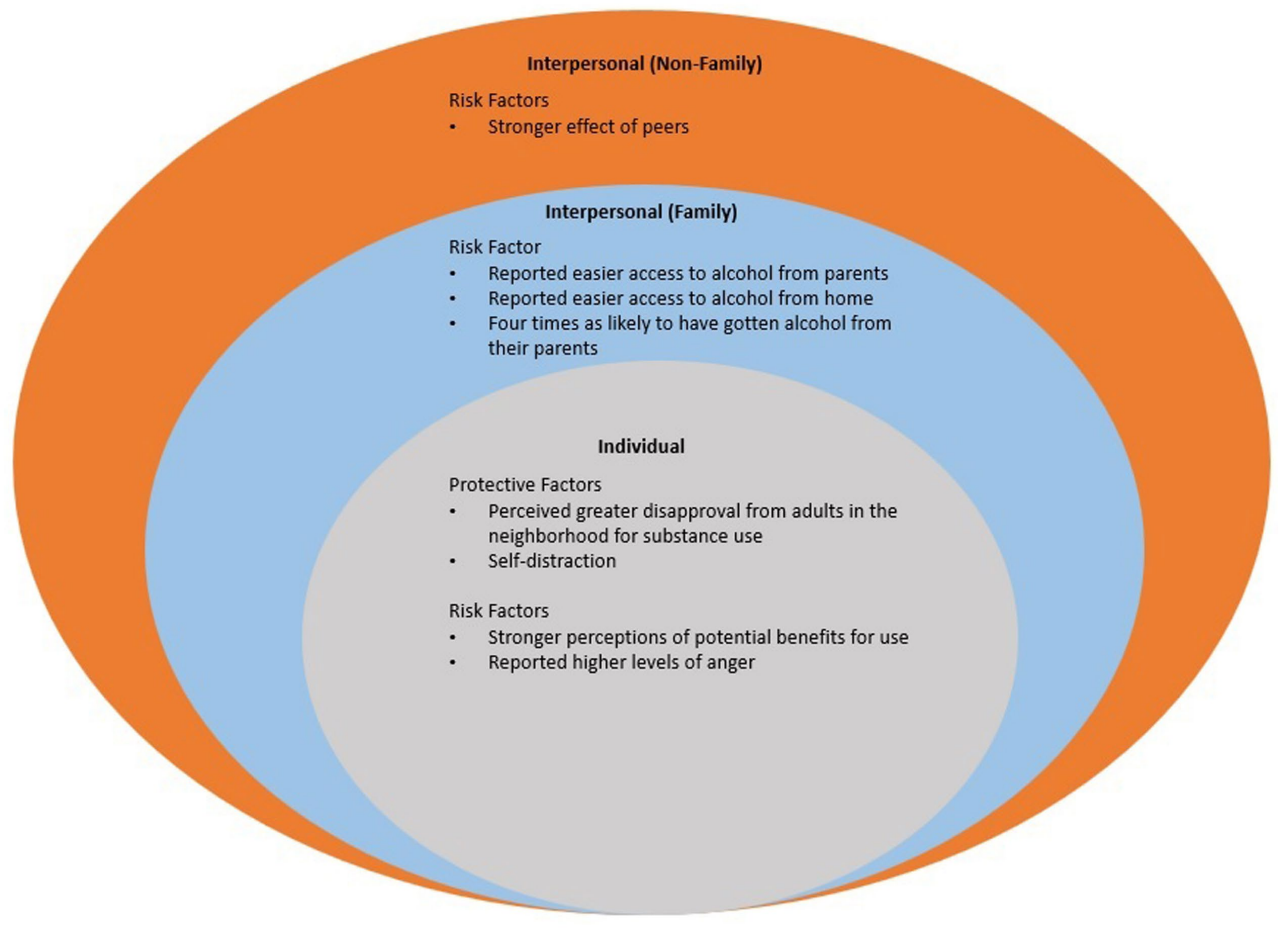
Risk and protective factors unique to white youth across the ecological model.

A total of 29 risk and protective factors unique to American Indian youth (*n* = 21) and White American youth (*n* = 8) presented across the SEM (see Figures 3, 4).

### Risk factors unique to American Indian and White American youth

American Indian youth were more likely to initiate substance use at a younger age (23, 24) and were twice as likely to get alcohol from an adult and someone under 21 (25). Greater substance use among American Indian youth was associated with living on or near reservations (26), lower school involvement (27), self-distraction (28), anxiety (29), and low self-esteem (30). More specifically, American Indian youth males thought hereditary factors and fate played a greater role in alcohol use (31). American Indian youth also reported easier access to alcohol in general (25, 32), lower parental disapproval for substances (33), lower levels of parental monitoring (34), greater neighborhood disorganization (27), lower police enforcement (27), weaker perceived anti-drug norms (27), and a stronger effect of weak peer injunctive norms (lack of disapproval) (33). The effect of peers (29, 35) and perceptions of potential benefits for use were stronger for White American youth (33). White American youth reported easier access to alcohol from parents and home (25), high levels of anger (29, 30).

### Protective factors unique to American Indian and White American youth

American Indian youth were more likely to perceive cigarettes as harmful (36) and were more likely to rate alcoholism as an illness/disease (37). American Indian youth were also less likely to get substances from parents (25) and home (32); and reported high levels of family caring (38). White American youth perceived greater disapproval from adults in the neighborhood for substance use (27) and self-distraction (28).

### Factors that were not unique to American Indian or White American youth

A multitude of risk and protective factor findings that were not unique (*n* = 55) to American Indian or White American youth remained. We highlight the similarities in risk and protective factors for each population, American Indian and White American youth, below.

### Similarities in risk factors between American Indian and White American youth

Similarities for risk factors included, alcohol use (39), stressful life events (28, 40) and stress exposure (40); greater approval for substances (36), perceptions favorable to substance use (23, 33, 35, 41); more perceived adult disapproval for substance use (24), outcome expectancies (28, 39); having a psychiatric diagnosis (39); and behavioral disengagement (28). Being in a romantic relationship (40), having friends who used inhalants (38), family member methamphetamine use (40) and family conflict (34) were also identified as similar risk factors for both ethnicities. Living in a county with more single-parent households and living in counties with higher median incomes were similar risk factors for both ethnicities at the community level (32).

### Similarities in protective factors between American Indian and White American youth

Protective factors that were similar for both ethnicities included coping strategies (42), negative attitudes and perceptions toward substance use (36), reasons for not using substances (danger to health and not interested) (23), and perceived adult disapproval (24). Both ethnicities attributed drinking primarily to the individual and external factors (distressing events, environment) as related influences on problem drinking (37). Peer encouragement (35), parental sanctions (34), family social support (40), students who had never tried inhalants reported higher levels of family caring and parental monitoring (38), higher parental median income (32), and living with both parents (38) were also identified as similar protective factors for both ethnicities. Students (both American Indian and White American) who never tried inhalants reported higher levels of school attachment (30). Living in a county with more American Indians was protective for alcohol accessibility for both ethnicities at the community level (32).

Note that some of the findings may be contradictory when comparing the “similarities in risk factors” to the “similarities in protective factors” for these populations. Through data extraction from the 19 relevant articles, multiple risk and protective factors were identified. The majority of the extracted data focused on risk factors. It was hypothesized and confirmed that most of the risk and protective factors found would be identified at the inner levels of the SEM (individual and interpersonal levels). It was also hypothesized and confirmed that unique differences would be identified between American Indian and White American youth. A total of 29 risk and protective factors unique to American Indian (*n* = 21) and White American (*n* = 8) youth presented across the SEM.

## Discussion

Multiple intervention programs, both evidence-based and promising-practices, exist at the individual, interpersonal (family and non-family), community and institutional levels of the SEM for youth in general, however culturally rooted evidenced-based programs specific to American Indian youth that are applicable across the SEM are often non-existent. Given the lack of investment and support for culturally rooted initiatives, one strategy that has been utilized to create culturally informed programming is the use of culturally adapted interventions, which maintain the core components of evidence-based practice but translate the intervention to be more relevant and consistent with the ideas, values, beliefs, norms, attitudes, and knowledge of the targeted population (21, 43). Although an option, this strategy is far from ideal. Tribal communities are calling for a “ground up” approach. Walters et al. shares exemplars of culturally grounded health intervention research and explains there are a multitude of Indigenist worldviews and protocols that are foundational to Native health interventions including: “(1) Original Instructions, (2) relational restoration, (3) narrative- [em]bodied transformation, and (4) Indigenist community-based participatory research (ICBPR) processes” (44). These strategies have been taken with community-based initiatives by informing the design and implementation of interventions that prioritize local Indigenous knowledges and have the potential to promote health-positive messages across all the levels of the SEM.

When addressing health disparities, another strategy is to focus on strength-based programming in American Indian youth populations in the United States. According to Rountree & Smith, “Central to this approach is the empowerment of the patient or client by focusing on inherent strengths, including both internal and external resources, rather than problems to be overcome” (45). Problems specific to American Indian communities are often the result of historical trauma and oppressive policies and practices (45). Substance use related protective factors can be utilized to implement strength-based programming in Tribal communities. Health promotion programs and activities are not a one-size fits all approach. It is beneficial to include culturally rooted programs to yield best results in American Indian communities.

One exemplar initiative that implemented an exploratory community-based research study to identify Tribal community sources of strength was the Native Transformations Project that took place in the Pacific Northwest (46). Utilizing a Tribal participatory approach, sources of strength were identified at the family, individual, community, and spiritual level for this Tribal community (46). Family sources of strength included: teachings; family, roles, rules, and rituals; protective parenting; ancestors; uncles; powerful women; and grandparents (46). Community sources of strength included: opportunities for learning and healing; social connections; strong elders; traditional laws; harvesting and sharing of resources; and healthy connections to the past (46). Individual sources of strength included: awareness; working on living; helping others; honoring one’s gift and speaking from the heart; power of the mind; and Indian name and being a namesake (46). Spiritual Sources of Strength included welcoming the spirit; belief in prayer; gatherings; warnings; rites of passage; and being on land and water (46). Overall, the authors described a rich array of Coast Salish sources of strength. All of these community identified protective factors were deemed as important to Tribal community participants and their wellness and recovery journey (46). Findings from the study were used to inform community based and culturally grounded interventions to reduce substance use disparities (46).

When creating a culturally rooted program, public health professionals should aim to improve their cultural awareness and ability to implement culturally safe practices when informing the design of an intervention. More broadly practitioners should aim to provide “culturally responsive, engaging, holistic, trauma-informed services to American Indian and Alaska Native clients” (21). In 2019, SAMHSA released the Treatment Improvement Protocol, TIP 61 titled “Behavior Health Services for American Indian and Alaska Natives” which summarizes substance use and discusses the importance of delivering culturally, responsive, evidence-based services. This resource presents culturally adapted resources as well as an American Indian framework outlining evidence-based tribal practices that can guide the development and implementation of behavioral health service evidence-based practices. Tip 61 includes a catalog of effective behavioral health practices for Tribal communities focusing on multiple areas including: community prevention and education; cultural and subsistence skill development; early intervention and skill building; individual and family treatment; and recovery services and supports. Also included are tools focused on integrative care, specifically approaches for the incorporation of traditional Tribal practices in behavioral health programs. This resource is designed for a broad audience, native and non-native professionals, and aims to improve practitioners understanding of Tribal communities and related colonial impacts, cultural considerations when working with Tribal communities, and program-level methods for achieving cultural responsiveness (21).

Whether culturally adapting or creating a culturally grounded evidence-based intervention, it is important that practitioners are knowledgeable and aware of the impacts of colonization; the importance of historical trauma; the role of culture and cultural identity; sovereignty; the significance of community; the value of family, the value of cultural awareness; the importance of taking a culturally responsive and strengths based approach; and most importantly, the diversity of Tribal communities and the unique presentation these considerations may take given the community a practitioner is working with.

In this systematic literature review, the comparison between American Indian and White American youth is examined to determine the similarities and differences in substance use related risk and protective factors across the SEM. Given culturally rooted evidence-based programs specific to American Indian youth that are applicable across the SEM are often non-existent, examination of factors unique to American Indian youth enables health practitioners to be responsive in their approach when determining how to structure health intervention programs that are informed by the risk and protective factor profiles for these populations. This approach could result in the development of an evidence base specific to and responsive to the needs of American Indian youth as well as the development of protective factor rich environments that are more inclusive.

## Strengths and limitations

This study included the use of Covidence, and in alignment with systematic review protocol, two reviewers independently voted on article significance without bias from the other reviewer and this reduced human error. Any conflicts between the two reviewers were examined and decided upon by the PI (author Nadeau).

A strength of this systematic literature review is the inclusion of articles from seven different databases with a search phrase that was inclusive of all relevant subject areas. Utilizing multiple databases expanded the breadth of the search and diversified the search strategy. Researchers who are interested in American Indian health can utilize the search process and method of presentation used in this review to identify and extract data for a multitude of health indicators. More broadly, the results of this literature review can identify potential research gaps to inform the direction of future research.

A limitation of this study is that there is a lack of research in risk and protective factor profiles for substance use comparing American Indian and White American youth. Additionally, there is a lack of data collection at local levels. The lack of research and existing literature, as well as the limited scope of the studies conducted, limited the authors’ ability to identify risk and protective factors across the social ecological model, specifically the institutional-, policy-, and cultural-level. Another limitation is that even with a broad search phrase and the utilization of multiple databases, there may have been relevant articles that were missed in the search process. And finally, a few of the unique differences identified for American Indian youth were identified prior to the year 2000 so this information may be considered dated. Researchers and community stakeholders should be cautious when using these findings and search for confirming evidence prior to designing potential interventions.

## Future research directions

Currently the American Indian population is underrepresented in research and surveillance. For example, the YRBSS (Youth Risk Behavior Surveillance System), is a national survey that monitors six categories of health-related behaviors, two of which are alcohol and other drug use and tobacco use (47). Although this survey gives researchers a snapshot of the mainstream majority youth populations living in the United States, it is not representative for American Indian youth. There are only two Tribal Nations, with representative samples, included in the survey: Cherokee Nation and the Winnebago Tribe (48). There is a need for more inclusiveness of Tribes across Indian country due to the vast geographic variation of Tribal populations as the current national snapshot is not representative of the 574 federally recognized Tribes (49). Data collection from urban Indian communities is even more sparse. This is important because, out of the 5.2 million American Indians and Alaska Natives, approximately 71% live in urban areas (20). Data captured from Tribal Nations and Urban Indian communities can drive public health spending decisions. If data from a community is not counted, its health needs go unrecognized and health care funding goes elsewhere. Another consideration, as previously noted, is the caution that should be used by researchers when using national datasets due to the vast geographical differences in health status that exist for Tribal Nations in the United States; since each Tribe is unique in population, language, land base, cultural practices, access to health care and resources.

Oversampling is also important for distinct American Indian populations. For example, the National Research Council (US) Committee on Population explains, “…we must oversample further if we desire separate estimates for individual Tribes (e.g., Navajo) or combined Tribes (e.g., Southwestern Indians)” (49). It is essential that researchers include enough American Indian individuals in a research study compared to the total number of American Indians living in that state where the research is being completed. For example, out of the total population in South Dakota, 12% of individuals identify as American Indian (3). Thus, in a research study population, researchers must include, at the minimum, 12% of those who identify as American Indian in the study population.

The amount of culturally informed and grounded research is also lacking across Indian country. More research is needed at local levels, especially in Tribal Nations across the United States as there is limited research in this field. More specifically, research is needed at the outer levels of the SEM (community-, institutional-, policy-, and cultural-levels). At the cultural level, research should ideally focus on culturally rooted protective factors. In using the SEM, the greatest impact for change comes from integrating multiple levels of the model. Nadeau et al. explains that cultural indicators span the SEM and, “Promoting culture and initiatives grounded in cultural values would be a meaningful way for Tribal communities to advocate, support and engage in protective factor rich environments and positively impact the health of youth at multiple levels of community” (50). To properly assess American Indian youth health and wellbeing, we must also look at conditions that create or limit opportunity. This will contribute an important perspective for understanding both the nature and the sources of disparate health outcomes and will guide viable and effective solutions (51). More broadly, future research could examine how risk and protective factors differ through a Social Determinants of Health lens and further explore combined approaches that are inclusive of efforts aligning with Indigenous environmental, conservation, social justice and climate change efforts. Regardless of which level a researcher is working at, they should ultimately be responsive to Tribal communities’ call to action for utilizing a culturally rooted approach when prioritizing local Indigenous knowledges and promoting health-positive messages because such an approach will impact all the levels of the SEM.

## Data availability statement

The original contributions presented in the study are included in the article/Supplementary material, further inquiries can be directed to the corresponding author.

## Author contributions

MN, KW, and DO: study conception and design of work. MN, KW, RS, and DO: data collection. MN, KW, and RS: data analysis and interpretation of results. MN and KW: draft manuscript preparation. All authors contributed to the article and approved the submitted version.
