# Supplementary material for: A comparison of the substance use related risk and protective factor profiles for American Indian and White American youth: a mixed studies review
Source: Front Public Health. 2024 Jan 31;12:1046655. doi: 10.3389/fpubh.2024.1046655 (PMC10864645; doi:10.3389/fpubh.2024.1046655)
Supplement: Supplementary file 1 [file Table_1.DOCX]

APPENDIX

**Table 1: Risk Factors for Substance Use**

|  | **Authors, year** | **AI and White Study Population** | **Location** | **Study Type, Instrument** | **Substance Type** | **Risk Factors** |
| --- | --- | --- | --- | --- | --- | --- |
| **Individual** | Albers, E. C., Santangelo, L. K., McKinlay, G., Cavote, S., & Rock, S. L. (2002). | Total: 628 (117 American Indian [14%], 511 White [62%]) | Nevada | Cross-sectional study, prevention service program | Alcohol, cigarettes, and other drugs | High school students (American Indian and White) have much greater approval for alcohol cigarettes and other drugs compared to elementary and middle schoolers |
| **Individual** | Beauvais, F., Wayman, J. C., Jumper-, P., Plested, B., & Helm, H. (2002). | Total: 11,698 participants from each ethnic sample (American Indian [33.3%], and Non-Hispanic White [33.3%]) | United States | Cohort study, survey | Inhalants | American Indian and White youth who reported low levels of perceived harm from occasional use of inhalants were more likely to be involved in inhalant use |
| **Individual** | Cockerham, W. C., Forslund, M. A., & Raboin, R. M. (1976). | Total: 511 youth (391 White [77%] and 120 American Indian [23%]) | Wyoming | Cross-sectional study, survey | Marijuana and other drugs | Significantly higher proportion of American Indian youth feel that in general it is all right for people to use drugs compared to White youth. Significantly higher proportion of American Indian youth tried marijuana at a younger age compared to White youth. Significantly higher proportion of American Indian youth think that their friends have favorable attitudes toward both marijuana and other drugs compared to White youth. Reasons for using other drugs and marijuana were similar in both American Indian and White youth including: “I enjoy it" and “I like the physical feeling of getting high” |
| **Individual** | Dieterich, S. E., Stanley, L. R., Swaim, R. C., & Beauvais, F. (2013). | Total: 2,787 youth (1,633 American Indians [59%] and 1,154 White [41%]) | Northwest, Northern Plains, Northeast, Southeast, Southern Great Plains, and Southwest | Cross-sectional study, American Drug and Alcohol Survey | Alcohol | Descriptive norms (perceptions about the prevalence of behavior in a particular population [what others are doing]) and the outcome expectancy of benefits to self were positively related to alcohol use (last-month alcohol use, last- month drunkenness, and binge drinking) for both American Indian and White youth. |
| **Individual** | Dieterich, S. E., Swaim, R. C., & Beauvais, F. (2013). | Total: 2,334 youth (984 American Indian [42%] & 1,350 White [58%]) | Northwest, Northern Plains, Northeast, Southeast, Southern Great Plains, and Southwest | Cross-sectional, American Drug and Alcohol Survey | Marijuana and inhalants | When perceptions of peer use are low, White youth perceptions of potential benefits for use are stronger influences compared to American Indian youth. American Indian youth inhalant use was higher when strong descriptive norms (perceptions about the prevalence of behavior in a particular population [what others are doing]) for use were paired with either low peer injunctive norms (what others think of a behavior) or high positive outcome expectancies |
| **Individual** | Eitle, D. J., & McNulty Eitle, T. (2013). | Total: 573 youth (113 American Indians [20%] & 460 White [80%]) | United States (rural state) | Cross-sectional study, Rural teen stress and health survey | Methamphetamine | Recent stressful life events and stress exposure were a statistically significant predictor of year and lifetime methamphetamine use for both ethnic groups |
| **Individual** | Federman, E. B., Costello, E. J., Angold, A., Farmer, E. M. Z., & Erkanli, A. (1997). | Total: 449 youth (341 White [76%] & 108 American Indian [24%]) | North Carolina | Epidemiologic Study, Great Smoky Mountains Study | Cigarette, smokeless tobacco, alcohol, and other drugs | Alcohol use and having a psychiatric diagnosis was associated with increased risk of later tobacco and illicit drug use for both American Indian and White youth |
| **Individual** | Friese, B., & Grube, J. (2008). | Total: 2,096 youth (361 American Indian [17%] & 1,735 White [83%]) | Wisconsin | Cross-sectional, surveys (Center for Substance Abuse Prevention)) | Alcohol | American Indian youth reported easier access to alcohol in general (parties, siblings, older persons, bars, and stores) compared to White youth. American Indian youth perceived it to be easier to get alcohol from stores and bars than White youth. |
| **Individual** | Friese, B., Grube, J. W., & Seninger, S. (2015). | Total:  4,942 (68.4% American Indians & 31.6% Whites) | Northwest, Northern Plains, Northeast, Southeast, Southern Great Plains, and Southwest | Cross-sectional, survey (Prevention Needs Assessment Community Student Survey, PNA) | Marijuana, tobacco, and alcohol | American Indian youth risk factors include lower school involvement, weaker perceived anti-drug norms, greater perceived neighborhood disorganization, and lower levels of perceived police enforcement compared to White youth |
| **Individual** | Friese, B., Grube, J. W., Seninger, S., Paschall, M. J., & Moore, R. S. (2011). | Total: 18,916 (1,416 American Indians [7%] & 17,500 Whites [93%]) | Montana | Cross-sectional, PNA survey (Prevention, Needs Assessment Community Student Survey) | Alcohol | American Indians were more likely to get alcohol from other social sources or from a store or shop |
| **Individual** | McNulty Eitle, T., & Eitle, D. (2014). | Total: 568 youth (72.4% White & 20.4% American Indian) | United States (rural state) | Cross-sectional, self-administered questionnaire | Marijuana and alcohol use | Behavioral disengagement (giving in or reducing efforts in difficult situations) was found to be a risk factor for substance use for both ethnic groups. Self-distraction was found effective for reducing marijuana in White youth, but increased marijuana use in American Indian youth. Age and being exposed to more recent life events were risk factors for both American Indian and White youth |
| **Individual** | Oetting, E. R., Swaim, R. C., Edwards, R. W., & Beauvais, F. (1989). | Total: 851 youth (327 American Indian [38%] & 524 White [62%]) | United States (Midwest) | Cross sectional, school-based survey | 11 drug types (alcohol, marijuana, uppers, downers, tranquilizers, quaaludes, heroin, cocaine, inhalants, PCP, and LSD) | Highest correlation for drug use in American Indian youth is anxiety. Highest correlation to alcohol and drugs for White youth is anger |
| **Individual** | Sage, G. P., & Burns, G. L. (1993). | Total: 106 youth (53 American Indian [50%] & 53 White [50%]) | Northwestern Montana | Cross-sectional, survey (Beckman’s rating scale) | Alcohol | American Indian youth males thought hereditary factors and fate played a greater role in alcohol use compared to other groups and rated environmental events as less important causes than White male youth |
| **Individual** | Swaim, R. C. (2015). | Total: 856 youth (683 American Indian [80%] & 173 White [20%]) | Northwest, Northern Plains, Northeast, Southeast, Southern Great Plains, and Southwest | Cross-sectional, school-based surveys | Inhalants | Lower self-esteem related to earlier initiation only in American Indian youth compared to White youth. White youth reported higher levels of anger among those who initiated inhalant use at age 13 or older |
| **Individual** | Swaim, R. C., Stanley, L. R., & Beauvais, F. (2013). | Total: 975 youth (497 American Indian [51%] & 478 White [49%]) | Washington, Oregon, Montana, Arizona, North Dakota, South Dakota, Minnesota, and Wisconsin | Cross-sectional, American Drug and Alcohol Survey | Alcohol, marijuana, and inhalants | 8^th^ grade American Indian students perceive more prevalence of drinking and less disapproval of drinking by other students compared to White youth. This disappeared at grade 10 and turned around at grade 12 (increased risk for social influence on drinking at younger ages compared to White students). American Indian youth perceived less disapproval of marijuana and inhalant use by classmates and adults compared to White youth |
| **Interpersonal (Family)** | Dieterich, S. E., Swaim, R. C., & Beauvais, F. (2013). | Total: 2,334 youth (984 American Indian [42%] & 1,350 White [58%]) | Northwest, Northern Plains, Northeast, Southeast, Southern Great Plains, and Southwest | Cross-sectional, American Drug and Alcohol Survey | Marijuana and inhalants | American Indian youth reported lower parental disapproval of substance use which increased risk for marijuana use compared to White youth. |
| **Interpersonal (Family)** | Eitle, D. J., & McNulty Eitle, T. (2013). | Total: 573 youth (113 American Indian [20%] & 460 White [80%]) | United States (rural state) | Cross-sectional, Rural teen stress and health study survey | Methamphetamine | Family member methamphetamine use was a risk for both American Indian and White youth |
| **Interpersonal (Family)** | Friese, B., & Grube, J. (2008). | Total: 2,096 youth (361 American Indian [17%] & 1,735 White [83%]) | Wisconsin | Cross-sectional, surveys (Center for Substance Abuse Prevention (CSAP)) | Alcohol | White youth are four times as likely to have gotten alcohol from their parents compared American Indian youth. White youth reported easier access to alcohol from parents and home compared to American Indian youth. |
| **Interpersonal (Family)** | Swaim, R. C., & Stanley, L. R. (2016). | Total: 4,942 youth (68.4% American Indian & 31.6% White) | Northwest, Northern Plains, Northeast, Southeast, Southern Great Plains, and Southwest | Cross-sectional, school-based survey | Marijuana | Family conflict is a risk for marijuana for both American Indian and White youth. American Indian students reported lower levels of parental monitoring compared to White youth |
| **Interpersonal (Non-Family)** | Beauvais, F., Wayman, J. C., Jumper-, P., Plested, B., & Helm, H. (2002). | Total: 11,698 youth from each ethnic sample (33% American Indian and 33% Non-Hispanic White) | United States | Cohort study, surveys | Inhalants | Effect of peers was stronger in the White population. Effect was greatest for White students for degree to which friends use inhalants and weakest among American Indian students |
| **Interpersonal (Non-Family)** | Dieterich, S. E., Swaim, R. C., & Beauvais, F. (2013). | Total: 2,334 youth (984 American Indian [42%] & 1,350 White [58%]) | Northwest, Northern Plains, Northeast, Southeast, Southern Great Plains, and Southwest | Cross-sectional, American Drug and Alcohol Survey | Marijuana and inhalants | The effect of weak peer injunctive norms (i.e., lack of disapproval) is stronger for American Indian youth |
| **Interpersonal (Non-Family)** | Eitle, D. J., & McNulty Eitle, T. (2013). | Total: 573 youth (113 American Indian [20%] & 460 White [80%]) | United States (rural state) | Cross-sectional, Rural teen stress and health study survey | Methamphetamine | Respondents in romantic relationships were more likely to use meth for both American Indian and White youth |
| **Interpersonal (Non-Family)** | Friese, B., & Grube, J. (2008). | Total: 449 youth (341 White [76%] and 108 American Indian [24%]) | Wisconsin | Cross-sectional, Center for Substance Abuse Prevention survey | Alcohol | American Indian youth were almost twice as likely to have gotten alcohol from an adult and twice as likely to have obtained alcohol from someone under 21 compared to White youth. |
| **Interpersonal (Non-Family)** | Oetting, E. R., Swaim, R. C., Edwards, R. W., & Beauvais, F. (1989). | Total: 851 youth (327 American Indian [38%] and 524 White [62%]) | United States (Midwest) | Cross-sectional, school-based surveys | 11 drug types (alcohol, marijuana, uppers, downers, tranquilizers, quaaludes, heroin, cocaine, inhalants, PCP, and LSD) | Peer alcohol associations are higher in White youth compared to American Indian youth (alcohol use in White youth mostly happens with peers) |
| **Interpersonal (Non-Family)** | Swaim, R. C. (2016). | Total: 5,094 youth (3,498 American Indian [67%] & 1,596 White [33%]) | Northwest, Northern Plains, Northeast, Southeast, Southern Great Plains, and Southwest | Cross-sectional, school-based surveys | Inhalants | Students who had tried inhalants reported more friends also using inhalants in both American Indian and White participants |
| **Community** | Friese, B., Grube, J. W., Seninger, S., Paschall, M. J., & Moore, R. S. (2011). | Total: 18,916 youth (1,416 American Indian [7%] & 17,500 White [93%]) | Montana | Cross-sectional, survey (PNA) | Alcohol | Living in a county with more single-parent households was positively related to alcohol use for both American Indian and White youth. Both American Indian and White youth living in counties with higher median incomes were more likely to report getting alcohol from home without permission, buying alcohol with a fake ID, and taking alcohol from a shop |
| **Community** | Stanley, L. R., & Swaim, R. C. (2015). | Total: 3,480 youth (2,497 American Indian [72%], 742 White [21%], 65 both American Indian and White [2%], and 121 American Indian and some other ethnicity [5%]) | Upper Great Lakes, Northern Plains, and Southwest Region | Cross-sectional study, school-based survey | Alcohol, marijuana, and inhalants | American Indian youth living on or near reservations initiate substance use earlier than White youth. Marijuana initiation ranged from 7-10x greater for 9 vs 8-year-old American Indian youth vs White youth. |
