# Supplementary material for: A comparison of the substance use related risk and protective factor profiles for American Indian and White American youth: a mixed studies review
Source: Front Public Health. 2024 Jan 31;12:1046655. doi: 10.3389/fpubh.2024.1046655 (PMC10864645; doi:10.3389/fpubh.2024.1046655)
Supplement: Supplementary file 2 [file Table_2.DOCX]

APPENDIX

**Table 2: Substance Use Protective Factors**

| **SEM Level** | **Authors, year** | **AI and White Study Population** | **Location** | **Study Type, Instrument** | **Substance Type** | **Protective Factors** |
| --- | --- | --- | --- | --- | --- | --- |
| **Individual** | Albers, E. C., Santangelo, L. K., McKinlay, G., Cavote, S., & Rock, S. L. (2002). | Total: 628 (117 American Indian [14%], 511 White [62%],) | Nevada | Cross-sectional study, prevention service program | Alcohol, cigarettes, and other drugs | White and American Indian youth had negative attitudes toward alcohol, cigarettes, and drug use. Whites find alcohol usage somewhat wrong. American Indian youth find drug usage somewhat wrong. Whites and American Indians perceive alcohol as harmful. American Indian students perceived cigarettes as harmful more than Whites. Elementary aged students were much more likely to perceive cigarette smoking as very harmful compared to older students for both American Indian and White youth |
| **Individual** | Cockerham, W. C., Forslund, M. A., & Raboin, R. M. (1976). | Total: 511 youth (391 White [77%] and 120 American Indian [23%]) | Wyoming | Cross-sectional study, survey | Marijuana and other drugs | The primary reasons for not using drugs other than marijuana were similar for both American Indian and White youth including “danger to health” and “not interested” |
| **Individual** | Dieterich, S. E., Swaim, R. C., & Beauvais, F. (2013). | Total: 2,334 students (984 American Indian [42%] and 1350 White [58%]) | Northwest, Northern Plains, Northeast, Southeast, Southern Great Plains, and Southwest | Cross-sectional study, American Drug and Alcohol Survey | Marijuana and inhalants | Perceived peer disapproval of inhalant use was higher for American Indian youth |
| **Individual** | Eitle, D., & McNulty Eitle, T. (2015). | Total: 568 (72.4% White and 20.4% American Indian) | United States (rural state) | Cross-sectional study, self-administered questionnaire | Marijuana and alcohol use | Problem-focused coping strategies (active and planning coping as well as instrumental coping) protective for substance use for Whites and American Indians. Also found religious coping strategies to be protective for substance use for both American Indians and White youth |
| **Individual** | Friese, B., Grube, J. W., & Seninger, S. (2015). | 13,224 youths (719 American Indians [5%] and 12,505 Whites [95%]) | Montana | Cross-sectional study, survey (Prevention Needs Assessment (PNA) Community Student Survey) | Marijuana, tobacco, and alcohol use | White youth perceived greater disapproval from adults in the neighborhood for substance use compared to American Indian youth |
| **Individual** | Jones-Saumty, D. J., Dru, R. L., & Zeiner, A. R. (1984). | Total: 165 (65 American Indian [39%] & 100 Whites [61%]) | Oklahoma | Cross-sectional study, Beckman’s rating scale | Alcohol | American Indian students rated alcoholism as an illness/disease significantly higher than White group. Both groups attributed problem drinking primarily to the individual and external factors (distressing events, environment) as concomitant influences on problem drinking |
| **Individual** | Swaim, R. C. (2015). | Total: 856 (683 American Indians [80%] & 173 Whites [20%]) | Northwest, Northern Plains, Northeast, Southeast, Southern Great Plains, and Southwest | Cross-sectional, school-based surveys | Inhalants | Students (both American Indian and White) who never tried inhalants reported higher levels of school attachment. |
| **Individual** | Swaim, R. C., Stanley, L. R., & Beauvais, F. (2013). | 975 youth (497 American Indian [51%] and 478 White [49%]) | Washington, Oregon, Montana, Arizona, North Dakota, South Dakota, Minnesota, and Wisconsin | Cross-sectional, school-based study | Alcohol, marijuana, and inhalants | 12^th^ grade American Indians and White females reported more perceived adult disapproval for alcohol, marijuana, and inhalants |
| **Interpersonal (Family)** | Eitle, D. J., & McNulty Eitle, T. (2013). | Total: 573 youth (113 American Indians [20%] & 460 White [80%]) | United States (rural state) | Cross-sectional study, Rural teen stress and health survey | Methamphetamine | Family social support protective for both American Indian and White youth for methamphetamine use |
| **Interpersonal (Family)** | Friese, B., & Grube, J. (2008). | Total: 2,096 youth (361 American Indian [17%] & 1,735 White [83%]) | Wisconsin | Cross-sectional, surveys (Center for Substance Abuse Prevention (CSAP)) | Alcohol | American Indian youth perceived it to be more difficult to get alcohol from parents. |
| **Interpersonal (Family)** | Friese, B., Grube, J. W., Seninger, S., Paschall, M. J., & Moore, R. S. (2011). | Total: 18,916 (1,416 American Indian [7%] & 7,500 White [93%]) | Montana | Cross-sectional study, survey (Prevention Needs Assessment (PNA) Community Student Survey) | Alcohol | American Indian youth less likely than White youth to get alcohol from their home. Household median income was negatively related to lifetime drinking and how easy it was to obtain alcohol for both American Indian and White youth |
| **Interpersonal (Family)** | Swaim, R. C. (2016). | Total: 5,094 (3,498 American Indians [69%], 1,596 Whites [31%]) | Northwest, Northern Plains, Northeast, Southeast, Southern Great Plains, and Southwest | Cross-sectional, school-based survey | Inhalants | Students who had never tried inhalants reported higher levels of family caring and parental monitoring. American Indian students reported higher levels of family caring but greater disparity in family caring between early and later initiators in White youth. Living with both parents was associated with a lower likelihood of lifetime and current marijuana use for both American Indian and White youth. |
| **Interpersonal (Family)** | Swaim, R. C., & Stanley, L. R. (2016). | Total:  4,942 (68.4% American Indians, 31.6% Whites) | Northwest, Northern Plains, Northeast, Southeast, Southern Great Plains, and Southwest | Cross-sectional, school-based survey | Marijuana | Living in a 2-parent household protective for marijuana (stronger for White youth). Parental sanctions protective for both groups. |
| **Interpersonal (Non-Family)** | Beauvais, F., Wayman, J. C., Jumper-, P., Plested, B., & Helm, H. (2002). | Total: 11,698 participants from each ethnic sample (American Indian [33.3%], and Non-Hispanic White [33.3%]) | United States | Cohort study, survey | Inhalants | Strong effect from peer encouragement was seen for both American Indian and White students |
| **Community** | Friese, B., Grube, J. W., Seninger, S., Paschall, M. J., & Moore, R. S. (2011). | Total: 18,916 (1,416 American Indians [7%] & 17,500 Whites [93%]) | Montana | Cross-sectional, PNA survey (Prevention, Needs Assessment Community Student Survey) | Alcohol | Living in a county with more American Indians reduced access to alcohol for both American Indian and White youth as well as reduced lifetime, 30-day drinking, heavy episodic drinking. |
