# Supplementary material for: A comparison of the substance use related risk and protective factor profiles for American Indian and White American youth: a mixed studies review
Source: Front Public Health. 2024 Jan 31;12:1046655. doi: 10.3389/fpubh.2024.1046655 (PMC10864645; doi:10.3389/fpubh.2024.1046655)
Supplement: Supplementary file 3 [file Table_3.DOCX]

SUPPLEMENTARY MATERIALS

**Sample search phrase: used to search PubMed**

("Substance-Related Disorders"[Mesh] OR "drug offense" OR "drug abuse" OR "drug misuse" OR "drug dependence" OR "drug addiction" OR "Drug Users"[Mesh] OR "substance use" OR "substance abuse" OR "substance misuse" OR "substance dependence" OR "substance addiction" OR "prescription abuse" OR "Alcoholism"[Mesh] OR "cannabis use disorder" OR "alcohol use disorder" OR "stimulant use disorder" OR "hallucinogen use disorder" OR "opioid use disorder" OR "inhalant use disorder" OR alcohol OR "Alcoholic Beverages"[Mesh] OR cannabis OR Marijuana OR "Cannabis"[Mesh] OR "Marijuana Abuse"[Mesh] OR "Marijuana Smoking"[Mesh] OR ((opioids OR "Narcotics"[Mesh] OR "Analgesics, Opioid"[Mesh] OR kratom OR hallucinogens OR "Psychotropic Drugs"[Mesh] OR inhalants OR toluene OR ((amyl OR butyl OR isobutyl) AND nitrites) OR stimulants OR "Central Nervous System Stimulants"[Mesh] OR "Amphetamines"[Mesh] OR Sedatives OR "Hypnotics and Sedatives"[Mesh] OR Benzodiazepines OR "Benzodiazepines"[Mesh] OR Anthramycin OR Bromazepam OR Clonazepam OR Devazepide OR Diazepam OR Flumazenil OR Flunitrazepam OR Flurazepam OR Fentanyl OR Alprazolam OR Clonidine OR Hashish Clonidine OR Lorazepam OR Nitrazepam OR Oxazepam OR Pirenzepine OR Prazepam OR Temazepam OR Chlordiazepoxide OR Clorazepate Dipotassium OR Estazolam OR Medazepam OR Midazolam OR Triazolam OR opioid* OR opiate* OR Heroin OR opium OR "Morphine Derivatives"[Mesh] OR Codeine OR Hydrocodone OR Oxycodone OR Dihydromorphine OR Ethylmorphine OR Heroin OR Hydromorphone OR Morphine OR Oxymorphone OR Thebaine OR Cocaine OR "Cocaine"[Mesh] OR Methamphetamine* OR "Methamphetamine"[Mesh] OR Benzphetamine OR anabolic steroids OR "Testosterone Congeners"[Mesh] OR antihistamines OR nitrous oxide OR betel nut OR kava OR Ecstasy OR phenylalkylamines OR mescaline OR 2,5-dimethoxy-4-methylamphetamine OR MDMA OR 3,4-methylenedioxymethamphetamine OR indoleamine* OR psilocybin OR psilocin OR dimethyltryptamine OR ergoline* OR lysergic acid diethylamide OR "morning glory seeds" OR "Salvia divinorum" OR jimsonweed OR anxiolytic OR benzodiazepine* OR zolpidem OR zaleplon OR carbamate* OR glutethimide OR meprobamate OR barbiturate* OR secobarbital OR barbiturate* OR glutethimide OR methaqualone OR amphetamine OR dextroamphetamine OR methamphetamine OR gabapentin OR baclofen OR diacetylmorphine OR kratom OR polydrug OR "poly-drug" OR polysubstance OR "poly-substance" OR "injection drug") AND (addict* OR abus* OR misus* OR disorder* OR mis-use OR dependen*)))

 ("Indians, North American"[Mesh] OR "Alaskan Natives"[Mesh] OR "Native American" OR "Alaska Native" OR "American Indian" OR "First Nations" OR Indigenous OR Métis OR Metis OR aborigin* OR "First Nation*" OR amerindian OR tribe OR tribal OR reservation OR "residential school" OR "urban indian" OR "Health Services, Indigenous"[Mesh] OR "United States Indian Health Service"[Mesh]) AND ("Whites"[Mesh])

("adolescent"[MeSH] OR adolescen* OR teen* OR "young people" OR "young person*" OR "young adult*" OR youth* OR girl OR girls OR boy OR boys OR juvenile* OR "Young Adult"[Mesh])

("Substance-Related Disorders"[Mesh] OR "drug offense" OR "drug abuse" OR "drug misuse" OR "drug dependence" OR "drug addiction" OR "Drug Users"[Mesh] OR "substance use" OR "substance abuse" OR "substance misuse" OR "substance dependence" OR "substance addiction" OR "prescription abuse" OR "Alcoholism"[Mesh] OR "cannabis use disorder" OR "alcohol use disorder" OR "stimulant use disorder" OR "hallucinogen use disorder" OR "opioid use disorder" OR "inhalant use disorder" OR alcohol OR "Alcoholic Beverages"[Mesh] OR cannabis OR Marijuana OR "Cannabis"[Mesh] OR "Marijuana Abuse"[Mesh] OR "Marijuana Smoking"[Mesh] OR ((opioids OR "Narcotics"[Mesh] OR "Analgesics, Opioid"[Mesh] OR kratom OR hallucinogens OR "Psychotropic Drugs"[Mesh] OR inhalants OR toluene OR ((amyl OR butyl OR isobutyl) AND nitrites) OR stimulants OR "Central Nervous System Stimulants"[Mesh] OR "Amphetamines"[Mesh] OR Sedatives OR "Hypnotics and Sedatives"[Mesh] OR Benzodiazepines OR "Benzodiazepines"[Mesh] OR Anthramycin OR Bromazepam OR Clonazepam OR Devazepide OR Diazepam OR Flumazenil OR Flunitrazepam OR Flurazepam OR Fentanyl OR Alprazolam OR Clonidine OR Hashish Clonidine OR Lorazepam OR Nitrazepam OR Oxazepam OR Pirenzepine OR Prazepam OR Temazepam OR Chlordiazepoxide OR Clorazepate Dipotassium OR Estazolam OR Medazepam OR Midazolam OR Triazolam OR opioid* OR opiate* OR Heroin OR opium OR "Morphine Derivatives"[Mesh] OR Codeine OR Hydrocodone OR Oxycodone OR Dihydromorphine OR Ethylmorphine OR Heroin OR Hydromorphone OR Morphine OR Oxymorphone OR Thebaine OR Cocaine OR "Cocaine"[Mesh] OR Methamphetamine* OR "Methamphetamine"[Mesh] OR Benzphetamine OR anabolic steroids OR "Testosterone Congeners"[Mesh] OR antihistamines OR nitrous oxide OR betel nut OR kava OR Ecstasy OR phenylalkylamines OR mescaline OR 2,5-dimethoxy-4-methylamphetamine OR MDMA OR 3,4-methylenedioxymethamphetamine OR indoleamine* OR psilocybin OR psilocin OR dimethyltryptamine OR ergoline* OR lysergic acid diethylamide OR "morning glory seeds" OR "Salvia divinorum" OR jimsonweed OR anxiolytic OR benzodiazepine* OR zolpidem OR zaleplon OR carbamate* OR glutethimide OR meprobamate OR barbiturate* OR secobarbital OR barbiturate* OR glutethimide OR methaqualone OR amphetamine OR dextroamphetamine OR methamphetamine OR gabapentin OR baclofen OR diacetylmorphine OR kratom OR polydrug OR "poly-drug" OR polysubstance OR "poly-substance" OR "injection drug") AND (addict* OR abus* OR misus* OR disorder* OR mis-use OR dependen* OR "harm reduction"))) AND ("Indians, North American"[Mesh] OR "Alaskan Natives"[Mesh] OR "Native American" OR "Alaska Native" OR "American Indian" OR "First Nations" OR Indigenous OR Métis OR Metis OR aborigin* OR "First Nation*" OR amerindian OR tribe OR tribal OR reservation OR "residential school" OR "urban indian" OR "Health Services, Indigenous"[Mesh] OR "United States Indian Health Service"[Mesh]) AND ("Whites"[Mesh]) AND ("adolescent"[MeSH] OR adolescen* OR teen* OR "young people" OR "young person*" OR "young adult*" OR youth* OR girl OR girls OR boy OR boys OR juvenile* OR "Young Adult"[Mesh])
